# Notes and Notices

**Published:** 1895-08

**Authors:** 


### NOTES AND NOTICES.

Dr. Wm. D. Gentry, whom we announced in the April number, at page 200, as having established a sanitarium at Fort Union, New Mexico, has been obliged, by adverse circumstances, to abandon that location and to remove to Las Vegas, New Mexico. This change of base has had the unfortunate effect of delaying the publication of the *Record of Materia Medica*. It will, however, be resumed before long, as he desires it to be a complete work before the opening of the new century.

Dr. M. R. Leverson, the Secretary of the Anti-Vaccination Society of America, is willing to make engagements for the delivery of lectures. He ought to be paid, but where funds are lacking he will lecture gratuitously. Thanks to the generosity of designer Weyprecht his lectures are illustrated. As Dr. Leverson is a highly successful lecturer on legislative science, social economy, and sanitation, arrangements might be made with reform clubs, economic circles, single-tax clubs, Y. M. C. A. lyceums, and mechanics' institutions, whereby expenses and receipts might be shared. His address is Port Richmond, Staten Island, New York.

Orificial Surgery.—Ninth annual course of instruction, by E. H. Pratt, A. M., M. D., LL. D., Professor of Orificial Surgery in the Chicago Homœopathic Medical College, Consulting Surgeon of Cook County Hospital, and Surgeon-in-Chief of the Pratt Sanatorium, will be held at the Chicago Homœopathic Medical College, September 2d, 3d, 4th, 5th, 6th, and 7th, 1895, corner of Wood and York Streets, Chicago.

The American Association of Orificial Surgeons will hold its eighth annual session at Apollo Hall, Central Music Hall, Chicago, September 4th and 5th, 1895, at 3 and 8 p. m. It promises to be the most interesting meeting of any in the history of the society. Arrangements have been made for practical papers by practical men. The programme is sufficient guarantee of a most profitable and interesting meeting.

As the Society is held in connection with Prof. Pratt's class in Orificial Philosophy, it affords a most opportune time for progressive physicians to renew their acquaintance with the subject, and learn of the results, not only from the master, but many of his followers.

You are cordially invited to be present and participate in its privileges.

You are also urged to use your influence in securing the attendance of all fellow-practitioners. This is a society for the modern doctor, and all are welcome.

The Homœopathic Medical Society, of the State of New York, will hold its forty-fourth semi-annual meeting at New York, October 1st and 2d, 1895, and its forty-fifth annual meeting at Albany, February 11th and 12th, 1896. President, Dr. Charles E. Jones, 140 State Street, Albany. Vice-Presidents, Drs. W. B. Gifford, of Attica; D. J. Roberts, New Rochelle; W. Louis Hartman, Clyde. Secretary, Dr. John L. Moffat, 17 Schermerhorn Street, Brooklyn; and Treasurer, Dr. Charles Deady, 110 West 48th Street, New York.

Southern Homœopathic Medical Association.—The next regular meeting of the Southern Association will be held in the city of St. Louis, in November, 1895. The meetings of this association have been growing in interest, value, and attendance, until now they rank next to the meetings of the American Institute. It is, perhaps, unnecessary to remind the profession of the benefit that will accrue from attendance on the annual gatherings of such a body of scientific medical workers, but it has been thought best to suggest that active participation in the transactions, by furnishing papers and by debating others that may be read, is a duty that should not be neglected. Next to and, in fact, equal with the live up-to-date professional information furnished by medical journals, is that obtained by attendance on medical societies, to say nothing of the fraternal and social pleasures enjoyed at the meetings.

No progressive, enlightened physician can afford to stay away from these meetings, and we confidently expect a large attendance.

The exact date of meeting, together with railroad and hotel rates, will be published in due time, and this reminder is only sent out at this early date to urge the members of the profession individually to commence now to make arrangements to come and to stimulate them to think up a topic for the paper to read. Wm. C. Richardson, M. D., President.

American Institute of Homœopathy.—Chicago, July 1st.—[Editor of *The Tribune.*]—The item in this morning's issue of *The Tribune*, relating to the American Institute of Homœopathy, is not true. The American Institute is in session at Newport, not Watch Hill. It did not elect Dr. Fink President. The meeting at Watch Hill was the International Hahnemannian Association, a small body of seceders from the American Institute, and composed of high potency, anti-vaccination cranks.—*H.*, *Chicago Tribune.*

The Woman's International Provers Association met at Newport and presented reports on provings of two drugs. That by the branch on the Pacific Coast, on Viburnum-opp., will be published in Trans. The proving of Ferr-iod. by the other members being incomplete, will not be published this year. The officers were re-elected and another drug chosen for work the coming year. Drs. Millie Chapman, President; Sophia Penfield, Secretary.

